# Exploring treatment burden in people with type 2 diabetes mellitus: a thematic analysis in china's primary care settings

**DOI:** 10.1186/s12875-024-02301-y

**Published:** 2024-03-15

**Authors:** Kai Lin, Mi Yao, Lesley Andrew, Rouyan Li, Yilin Chen, Jacques Oosthuizen, Moira Sim, Yongsong Chen

**Affiliations:** 1 Family Medicine Centre, The First Affiliated Hospital of Shantou University Medical College, Shantou, 515000 China; 2 School of Medical and Health Sciences, Edith Cowan University, Perth, 6027 Australia; 3 General Practice, Peking University First Hospital, Beijing, 100034 China; 4 School of Nursing and Midwifery, Edith Cowan University, Perth, 6027 Australia; 5 Clinical Medicine, Shantou University Medical College, Shantou, 515000 China; 6 Endocrinology Department, The First Affiliated Hospital of Shantou University Medical College, Shantou, 515000 China

**Keywords:** Diabetes Mellitus Type 2, Treatment burden, Focus group, Conceptual framework, Primary care settings

## Abstract

**Background:**

Understanding treatment burden is a critical element to the effective management of Type 2 Diabetes Mellitus (T2DM). The current study aims to address the knowledge gap surrounding treatment burden of T2DM from the patient’s perspective in China’s primary care settings.

**Methods:**

A narrative review informed the creation of an a priori coding structure to identify aspects of T2DM treatment burden. Focus groups were conducted, employing a maximum variation sampling strategy to select participants from diverse sociodemographic backgrounds across urban, suburban, rural, and remote areas in China. Participants included adults with T2DM care in primary care settings for over a year and a Treatment Burden Questionnaire score of 25 or higher. Deductive thematic analysis, guided by the coding structure, facilitated a comprehensive exploration and further development of the conceptual framework of T2DM treatment burden.

**Results:**

Four focus groups, each comprising five participants from diverse areas, were conducted. Utilising the Cumulative Complexity Model and Normalisation Process Theory as theoretical underpinnings, the thematic analysis refined the conceptual framework based on the coding structure from the narrative review. Five key themes were refined, encompassing medical information, medication, administration, healthcare system, and lifestyle. Additionally, the financial and time/travel themes merged into a new theme termed "personal resources", illustrating their overlapping within the framework. Participants in these focus groups highlighted challenges in managing medical information, an aspect often underrepresented in prior treatment burden research. The thematic analysis culminated in a finalised conceptual framework, offering a comprehensive understanding of the treatment burden experiences of people with T2DM in China’s primary care settings. This framework includes six key constructs, delineating T2DM treatment burden and associated factors, such as antecedents and consequences.

**Conclusions:**

This study provides insights into the treatment burden of T2DM. A conceptual framework was finalised to deepen the understanding of the multifaceted constructs and the nature of treatment burden in people with T2DM. Furthermore, it emphasises the need to tailor T2DM treatment to individual capacities, considering their personal resource allocation and treatment utilisation.

## Introduction

Type 2 Diabetes Mellitus (T2DM), representing over 90% of diabetes cases globally, poses a significant health challenge, with 541 million adults at risk of developing T2DM [1]. The complexity of T2DM treatment as an individual involves not just dealing with the effects of the disease, its complications, and medical treatment, but also coping with various strategies required to manage the disease. These tasks result in a substantial treatment burden, encompassing workload and costs that impact an individual’s behavioural, cognitive, physical, and psychosocial health [2, 3]. Adherence to T2DM treatments often demands substantial personal resources [4]. However, the lack of consideration of personal preference and the fragmented treatment focus exacerbate this burden [5]. Treatment burden is conceptualised as the workload that individuals perceive in managing their healthcare, which impacts various dimensions of health, such as behavioural, cognitive, physical, and psychosocial aspects [6]. Current evidence suggests that multiple subconstructs significantly influence the treatment burden for chronic diseases [7–9]. The Cumulative Complexity Model (CuCoM) presents a functional, patient-centred approach to understanding patient complexity, emphasising the balance between individuals’ workload of demands and patient capacity to address demands [10]. An imbalance, characterised by high healthcare demands surpassing the patient's limited capacity, exacerbates the treatment burden. This leads to a feedback loop, further perpetuating the cycle of increased burden [11]. A conceptual framework developed by Sav et al. integrates the various subconstructs of treatment burden, considering the dynamic interplay between these workloads and individual capacities [12].

Clinical practice for T2DM in China aligns with international guidelines [13]. However, there is typically a prevailing emphasis on hospital-based and specialist care models across the nation[14]. Over the last decade, China has faced major challenges in transitioning to a primary care-focused model. These challenges are particularly evident in the context of healthcare delivery transformation and the need for resource reallocation [15]. Additionally, there is a notable lack of supportive information and relevant research, especially in primary care and low-resource settings [16]. In our previous work, despite developing a systematic search strategy, limited qualitative research was retrieved from these settings [17]. The current study aims to explore the treatment burden experiences of individuals with T2DM within China’s primary care settings.

## Methods

### Study design

This study utilised thematic analysis within a pragmatism research paradigm [18–20]. A preliminary coding structure (Table 1), was developed through a comprehensive literature review, including narrative review contributions [21]. The a priori validated coding and themes expedited the conventional theme-searching process [22]. Data from focus group, comprising individuals with T2DM in China's primary care settings, was instrumental in refining this coding structure. This process further developed, described, and elaborated on the sub-themes and themes [20]. The final conceptual framework provides an interpretive lens for understanding the T2DM treatment burden [23].

**Table 1:** A preliminary coding structure

Themes	Sub-themes
Financial [key constructs]	Out-of-pocket expenses
	Implicit costs associated with treatment
Medication [key constructs]	Complexity of medication use
	Management of medications
	Drug dependence
	Side effect
Administrative [key constructs]	Challenges of medical regimen
	Documentation and paperwork
	Arranging appointments
Lifestyle [key constructs]	Challenges of health behaviours
	Change of nature behaviour
Healthcare [key constructs]	Health care fragmentation
	Health care provider obstacles
	Difficulty navigating the health system
	Insurance or recourse use
Time/travel [key constructs]	Transport difficulty
	Time spent
Medical information [key constructs]	Cumbersome medical information
	Lack of effective sources of information
	Biased information
Antecedents [associated factors]	People characteristics
	Disease characteristics
Consequences [associated factors]	Adherence to treatment
	Health and wellbeing and quality of life
	Interpersonal and social challenges
[emerged constructs]	Insulin- or injection-related burden
[emerged constructs]	Medication-related Hypoglycaemia
[emerged constructs]	Glucose meters

### Narrative review

The narrative review employed Boell’s hermeneutic approach [24, 25]. The initial literature review identified a conceptual scope of T2DM treatment burden and informed the development of search strategies [7–9]. The conceptual framework proposed by Sav et al. for measuring generic treatment burden guided both the identification of literature and the data synthesis [11, 12]. Literature from inception to April 2022 was searched in four English and three Chinese databases. The inclusion criteria were qualitative studies with a focus on the burdens in adults (> 18 years) undergoing T2DM treatment. Studies on disease burden, diabetes distress, and treatment satisfaction were excluded. Five qualitative and one mixed-methods study were included [26–31]. All the included studies were published in English. The reported data and findings in these studies were considered valid qualitative data. Two reviewers (K.L. and M.Y.) independently examined the studies. Data analysis and synthesis involved four steps: coding, sorting, synthesising, and theorising [32]. Subsequently, the codes were integrated into a coding structure. This analysis underwent review by a third party (L.A., J.O., Y.C., M.S.). The updated systematic review and comprehensive findings will be reported separately. This research is registered with the International Prospective Register of Systematic Reviews (PROSPERO, CRD42022244190) [17].

### Participants and settings

For the development of a further conceptual framework, individuals with T2DM were invited to participate in focus groups. From April to June 2022, these participants were recruited from primary care settings across China. In these focus groups, a purposive sampling strategy, based on the principle of maximum variation, was employed [33, 34]. This approach acknowledges that participants' perceptions of treatment burden are inherently influenced by their varying contexts [35]. Given the disparity in resource distribution in primary care within China, where coastal urban areas are typically more resource-rich than rural and remote inland areas [36, 37], this strategy specifically aimed to capture a broad spectrum of experiences in T2DM treatment burden.

Based on China’s urban–rural classification code, regions are generally classified as urban (code 111), suburban (code 121), and rural (code 210) areas [38]. In Guangdong Province, one region from each classification was selected, and an additional region (code 210) was chosen in Sichuan Province to represent a remote inland area. In each region, more than three primary care clinics were approached, with at least two in each region agreeing to participate. Participants were recruited from a national programme where individuals with diabetes or hypertension are routinely followed up in primary care settings and are required to register annually [39]. Flyers, both in physical and digital formats, were distributed in these clinics to introduce the research and invite eligible participants. The flyers included a QR code linking to the Chinese version of the Treatment Burden Questionnaire (TBQ) [40]. Eligibility criteria for participants included being adults aged 18 or older, having a diagnosis of T2DM for over a year, and having TBQ scores of 25 or higher out of 150. Those with cognitive or communicative impairments affecting their ability to effectively participate in group discussions were excluded.

K.L. contacted potential participants via telephone to present the study as a third-party investigation and coordinate focus group logistics. In each region, 1–2 individuals were unable to participate due to scheduling conflicts, and alternative participants with similar TBQ scores and demographic profiles were selected from the pool of potential candidates.

Ultimately, four focus groups were recruited, each from one of the selected regions [41]. Focus Group (FG) 1 was conducted in an urban area (Shenzhen, Guangdong), FG2 in a suburban area (Shantou, Guangdong), FG3 in a remote area (Chengdu, Sichuan), and FG4 in a rural area (Shaoguan, Guangdong). Each group convened at a primary care clinic in their respective region. Participants were offered RMB 200 (approximately USD $28) as compensation for travel expenses.

### Data Collection

Data collection was carried out from June to August 2022. All focus group sessions were organised and led by one researcher (K.L.), with each session lasting approximately 90 min. An experienced researcher in qualitative research and treatment burden (M.Y.) supervised these sessions and recorded field notes. Neither researcher had prior relationships with the participants.

During the focus groups, after presenting the introductory remarks and questions, the researchers adopted a non-participatory role. They refrained from engaging in discussion or responding to participants' questions, maintaining a neutral stance. Strictly adhering to the coding structure in focus groups can pose risks. Participants with in-depth knowledge might perceive rapid transitions between questions as abrupt, potentially leading to a loss of valuable insights not encompassed by the predefined themes [22]. Therefore, the focus group guide (Table 2) was designed with open-ended questions and supplemented by probing queries informed by the coding structure [42]. These approaches aimed to minimise potential biases and power dynamics between the researchers and the participants. All sessions were audio-recorded and transcribed verbatim for analysis.

**Table 2:** Focus group guide

Guide	Descriptions
1. Introduction to the Focus Group	Welcome to our focus group session. This meeting is part of our research study exploring the treatment burden experiences of people with Type 2 Diabetes Mellitus (T2DM) in primary care settings in China. T2DM is a prevalent condition globally, affecting a significant portion of the adult population. It involves a range of treatment and management tasks, leading to a substantial treatment burden that can impact various aspects of patients' lives, including their behavioural, cognitive, physical, and psychosocial health. Objective of the Focus Group Our focus today is to understand your experiences and challenges in the T2DM treatment. This includes the efforts you make to adhere to treatments and how these tasks affect your daily life. We aim to discuss the components involved in the treatment burden of T2DM and how it influences you. Demographic Information In this study, we aim to gather demographic information from participants in a manner that respects anonymity. This includes details such as gender, age group, duration of T2DM diagnosis in years, duration of follow-up visits in years, and location. This data will help us assess the representativeness of our sample relative to the broader population of individuals with T2DM.
2. Structure of the Session	The session will last approximately 90 min. We will present a series of questions related to your experiences with treatment burden of T2DM. You are encouraged to share your thoughts on each question, but you are not obligated to answer every question if you do not feel comfortable. There will be opportunities for open discussion, where you can interact with other participants and share your views. We would like to clarify that our aim is not to judge your responses. Our researchers will adopt a neutral, non-participatory role in the discussions. We will avoid interrupting your discussion or responding directly to any queries.
3. Guidelines for Participation	Confidentiality: Please be assured that your identity and responses will remain confidential. The information you share will be used solely for research purposes. Open Communication: We encourage open and honest communication. Feel free to express your thoughts and experiences without any hesitation. Respectful Interaction: Please be respectful of others' opinions and experiences. We value diverse perspectives and aim to foster a supportive environment. Voluntary Participation: Participation is entirely voluntary. You are free to withdraw at any point without any consequences.
4. Question Displayed:	The research question will be displayed on a screen throughout the focus group session for reference. Primary research question: " What are the characteristics of treatment burden experienced by people with T2DM in primary care settings?" Questions and Probes: (1) Characteristics of experienced Treatment Burden "What characteristics do you experience as part of your treatment burden in daily treatment of T2DM?" Probes: Can you identify specific aspects of your T2DM treatment that you find particularly burdensome? This could include components related to: ­-Financial Burden -­Medications ­-Administrative Tasks & Monitoring ­-Lifestyle Changes ­-Healthcare & Reimbursement System ­-Time & Travel Burden ­-Medical Information ­-etc. (2) Factors associated with Treatment Burden "From your perspective, what factors contribute to or are influenced by the burden of T2DM treatment?" Probes: Are there any Antecedents or Consequences of T2DM treatment burden? This might include components related to: -living with T2DM that either facilitate or impede your ability to manage T2DM treatment effectively, such as people or disease characteristics -health outcomes, such as adherence to treatment, health status, wellbeing, quality of life, interpersonal and social challenges

### Data management and analysis

All focus group discussions were primarily conducted in Chinese (Mandarin), with the inclusion of local dialects in rural and remote areas. Transcriptions were completed within 24 h by one researcher (K.L.). The transcripts were not returned to participants for comments. Instead, two researchers (R.L., Y.L.C.), who are knowledgeable in local culture and dialects, rigorously reviewed the transcriptions against the recordings. Additionally, the on-site supervisor (M.Y.) reviewed the final transcripts, providing feedback supplemented by field notes. For data management and analysis, MAXDQA 2020 software was employed by the research team.

The narrative review established a coding structure, which included seven key constructs: financial, medication, administrative, lifestyle, healthcare, time/travel, and medical information, each with sub-constructs (Table 1). The focus group analysis utilised deductive thematic analysis based on this coding structure [19, 20]. Four researchers (K.L., M.Y., Y.L.C., R.L.) independently coded the transcripts and integrated their findings into the software, paralleling the focus group sessions. After the first focus group (FG1), the team reviewed and coded the transcripts, engaging in discussions to reach a consensus on the coding. K.L. and M.Y. reviewed the codes and repeated readings of the transcripts, then collectively refined the coding structure, incorporating emergent components not initially identified. These agreed-upon thematic codes, marked as “[emerged constructs]” in Table 1, were incorporated into the structure, facilitating a comprehensive and in-depth analysis of the data [20]. Upon completing all focus groups, K.L. and M.Y. conducted a final review, merging, deleting, and refining themes and sub-themes to develop a finalised conceptual framework. This iterative process entailed ongoing team discussions until mutual agreement was reached, with no new theme emerging. It also involved a careful comparison with the preliminary coding structure. In translating the findings from Chinese to English, key nuances were preserved, ensuring the accuracy and fidelity of the thematic analysis to the original data. Validation of the findings was achieved through engaging experts in qualitative research and primary care (L.A. and M.S.) for in-depth discussions on the study's coding, themes and concepts.

### Reflexivity and trustworthiness

The study was conducted through collaborative interactions between researchers and participants, with the researchers keenly aware of the potential influence of their own backgrounds, beliefs, and biases, as well as those of the participants, on the study [20]. The data collection and analysis team comprised four researchers: two males and two females, all equal in their roles. K.L. and M.Y. both are general practitioners with PhD training in qualitative methodologies. Y.L.C. and R.L. are also general practitioners with extensive experience in primary care research in China. Throughout the research process, reflections on their thoughts, feelings, and potential biases were consistently documented. These records were regularly reviewed by the on-site facilitator (K.L.) to ensure objectivity and reflexivity. No instances of bias were identified during these reflexivity checks.

To ensure dependability, the research team engaged in regular peer debriefing sessions, cross-validating findings and interpretations during data analysis. This allowed for the thorough discussion of diverse perspectives. Sampling based on national standards for regional representativeness further enhances the transferability of our findings, making them relevant to various primary care settings across different regions in China.

### Patient and Public Involvement and Engagement (PPIE)

In addition, our research benefited from the contributions of a panel formed at a primary care clinic in China. This panel comprised four individuals with T2DM, a nurse, a public health doctor, a traditional medicine doctor, and a general practitioner. Two workshops were conducted with these panel members. Drawing upon their diverse experiences, they rigorously reviewed and provided feedback on the rationality of the coding structure and the clarity of the finalised conceptual framework, as well as the descriptions of themes. This approach encouraged active participation and contribution from the panel members, aiming to enhance the contextual relevance and practical value of the research findings.

### Ethical considerations

Ethical approval for this study was granted by the Human Research Ethics Committee of The First Affiliated Hospital of Shantou University Medical College (Approval No. B-2022–238) and the Edith Cowan University Human Research Ethics Committee (REMS No. 2021–03129-KA). Participant anonymity and confidentiality were ensured throughout the study. All participants provided written informed consent before participating and answering the questionnaires.

## Results

### Participant characteristics

All invited participants attended the focus groups, except for one (P1, FG1) who could not join due to a time conflict and was instead interviewed individually. Table 3 presents the demographics of the 20 participants. The age distribution included 11 participants over 65 years, seven between 40 and 65 years, and two under 40 years, with a balanced gender distribution. Regarding healthcare engagement, 12 participants had been following up in primary care settings for less than 3 years, 5 for 3–10 years, and 3 for over 10 years. The sampling represented a wide range of primary care settings located in diverse socio-economic regions across China.

**Table 3:** Participant characteristics (*n* = 20)

Subjects		Frequency	Percentage (%)
Age	< 40	2	10.00
	40 ~ 65	7	35.00
	> 65	11	55.00
Gender	female	10	50.00
	male	10	50.00
Duration of T2DM diagnosis (years)	< 5	11	55.00
	≥ 5	9	45.00
Duration of follow-up in primary care (years)	1 ~ 2	12	60.00
	3 ~ 10	5	25.00
	≥ 10	3	15.00
Location	urban (FG1)	5	25.00
	suburban (FG2)	5	25.00
	rural (FG4)	5	25.00
	remote (FG3)	5	25.00
Total		20	100.0

### Conceptual framework development

During the focus groups, five key constructs from the preliminary coding structure (medical information, medication, administration, healthcare system, and lifestyle) were further developed with subtheme clarification. The constructs of financial burden and time/travel burden exhibited overlapping nature, leading to their integration into a newly defined theme termed "personal resources". As a result, the finalised conceptual framework comprised six themes, encapsulating the essential constructs shaping the concept of T2DM treatment burden in primary care (Table 4).

**Table 4:** Finalised conceptual framework

**Themes**	**Sub themes**	**Description**	
**Medical Information**	**Cumbersome medical information**	The complexity and poor user-friendliness of medical information presentation	*" I still don't understand what high blood sugar means. The doctors just keep asking me to have my blood drawn to test it, to find out exactly how high it is…" (FG4, P1)* *"This confuses me, how can one disease have so many different therapies? … I think there might be different types of Metformin… it's different in the hospital (compared to what's available in community clinic or pharmacy near my suburb). " (FG4, P2)*
	**Lack of sources of information**	The struggle to find consistent and personalised medical information	*"Since being diagnosed, I've been paying close attention to this issue. I like to consult various doctors for advice, but I found that different doctors give different suggestions." (FG4, P1)* *"The doctor only gave me some general dietary advice. It doesn't really have anything to do with what I usually eat, like I don't have noodles. But I don't know how to look for other trustworthy information." (FG1, P2)*
	**Biased information**	This construct captures the impact of culturally and societally influenced information on the excessive treatment workload or burden. The challenge arises not from a lack of information but rather from cultural and societal distortions of the information available	*"I don't want to use insulin because it causes hypoglycaemia. I've been told that by others, and I know, I know that hypoglycaemia is horrible." (FG1, P3)* *"Another patient asked, 'Why is your hand that colour?' Answered: 'It's because of the medication. No, not exactly the diabetes medication. But I feel it is.' " (FG2, P1, P5)* *"I just stick to rice, you know? They say that's all we can have, and now it's pretty much all I eat," remarked a participant, expressing a prevalent dietary misconception among Chinese patients that eating only rice and vegetables is synonymous with health in their cultural context, specifically in remoted area (FG3, P2)* *"I tried using vinegar-soaked eggs, but it (preparation process) is too much trouble, and it doesn't seem to be very effective." (FG1, P3)*
**Healthcare System**	**Healthcare fragmentation**	The challenges arising from system fragmentation are substantial, where patients must navigate a segmented healthcare system and coordinate treatment across multiple departments and providers	*"Going to the hospital and figuring out which department to register with wastes a lot of time." (FG3, P2)* *"The most troublesome thing is that every time I go, there are different doctors, sometimes nurses. The varying advice confuses me." (FG4, P2)*
	**Healthcare provider**	The complex challenges in patient-provider interactions and consultations, emphasising constrains in the consultation and the impact of the healthcare providers' attitude	*"In the room, doctors consult with numerous patients, leaving me with limited time to communicate. Each visit to the doctor is tense (both in terms of time constraints and emotional stress)." (FG1, P4)* *"During my experience, an unpleasant attitude from healthcare staff really impacts my perceptions and experiences with treatment, primarily due to losing confidence." (FG3, P5)*
	**Insurance or recourse use**	The complexities of insurance and healthcare resource utilisation, such as inconsistent reimbursement processes and bureaucratic obstacles	*"Most of the time, insurance only pays for when you're actually in the hospital. Anything outside (outpatient clinic), you're on your own." (FG2, P5)* *"The rules for getting money back are all over the place. Different insurances make you jump through different hoops." (FG4, P4)*
	**Difficulty with healthcare access**	Emphasises systemic barriers in accessing healthcare services, influenced by hospital protocols, and external factors like pandemics	*"Why can't hospitals do the same? Like banks keep a couple of counters, just for us (follow-up patient), just make it simple." (FG3, P2)* *"Getting into the hospital is like going through airport security (during pandemic). You got to show your travel code, health code, and even your COVID test results. Then, there are lots of forms for all of it." (FG1, P2)*
**Administration**	**Periodic examination/monitoring**	The challenges associated with frequent medical check-ups and the resource-intensive nature of routine monitoring	*"They often ask us to repeatedly check our blood sugar, right? Only after examination can they prescribe medication. ……I feel like there are too few settings (in community). And every time I go to the hospital for a check-up, it's very troublesome, and I have to queue for a long time." (FG4, P2)* *"I have to go to the hospital (instead of the community clinic) every three months. Mainly to see specialists and for periodic examination, because there is a lack of machines in the community health facilities." (FG1, P1)*
	**Arranging appointments**	The difficulties faced in scheduling medical visits, especially by senior patients who may struggle with technological systems	*"Both seeing the doctor and getting prescriptions are burdensome. When I was first diagnosed, I had to see the doctor frequently." (FG4, P1)* *"Initially, I followed my usual procedure of going to the clinic. However, all the appointment slots were booked up (by others online) …… no slots remained available for elderly." (FG3, P1)*
	**Documentation and paperwork**	The complexities of keeping health records and remembering what needs to be done, in terms of compiling, updating, and maintaining the documents	*"My main burden is monitoring and recording. Sometimes I test my blood sugar, but I didn't record it. The doctor asked me to keep continuous records." (FG4, P1)* *"Yes, to test blood sugar, and then record it, and medicine intake, and dietary (all need to be kept in health record), or they will ask you more." (FG4, P4)*
	**Glucose meters**	The use of glucose meters in T2DM treatment poses specific administrative challenges, particularly regarding the need for consistent monitoring, the frequency of use, maintenance of the meter, and the discomfort associated with its use in self-management	*"The procedure requires checking blood sugar levels before meals using fingertip blood, and the needle scares me." (FG1, P1)* *"I need to buy needle every month. If the needle of the glucose meter has been stored for an extended period, I worry that it may lose its accuracy. " (FG2, P1)*
**Medication**	**Management of medications**	The logistical challenges in adhering to medication management, encompassing issues like medication storage, concerns about shelf-life, and the cognitive demands of various treatment schedules	*"Remembering to take my medication is a challenge. I often forget, especially when I'm busy." (FG1, P1)* *"Dealing with medication is a burden… remembering to take the medication is hard and carrying it around when going out is inconvenient." (FG4, P3)* *"Getting medication from the pharmacy is a hassle. Sometimes they don't have what I need." (FG3, P3)*
	**Complexity of medication use**	The cognitive load and stress from complex medication use regimens, such as the number of medications, timing, and potential interactions concern	*"Taking multiple medications at different times of the day is confusing. I sometimes mix up the times." (FG3, P1)* *"I used herbal medicine before, it smelled terrible." (FG2, P4)*
	**Ambivalence towards medication**	This construct encapsulates patients' mixed feelings about their diabetes medication, balancing the recognised necessity of these drugs for health management with concerns over dependency	*"If I don't take my medication, I feel anxious. It's like I'm addicted to it." (FG1, P4)*
	**Side effects and hypoglycaemia**	Deals with the management of medication side effects, particularly the risks and fears surrounding hypoglycaemia	*"I've experienced low blood sugar. It's terrifying because it feels even worse than high blood sugar.A friend told me to put a candy in my pocket and I've been keeping it in mind." (FG2, P5)*
	**Insulin- or injection-related burden**	The unique logistical and emotional issues related to insulin or injection therapies, such as concerns of usage, storage, or public stigma	*"Storing insulin while traveling is a problem because it needs refrigeration." (FG2, P6)* *"I feel embarrassed when injecting insulin in public places. I always have to find a private place to do it." (FG4, P2)*
**Lifestyle**	**Interruption of lifestyle and daily routines**	Highlights the lifestyle changes necessitated by T2DM treatment, such as dietary adjustments and change in leisure activities, emphasising the conflict between personal lifestyle choices and the compromises required by the disease	*"I used to enjoy travelling, but with the way I have to manage my condition now, I just don't have the courage to go far. I stick to places close by and then head back home." (FG2, P2)* *"Each morning follows a strict routine to comply with my health advice—monitoring my blood sugar, preparing a meal that fits my diabetes management plan, and administering insulin before I eat." (FG1, P3)* *"For example, I particularly like to eat lychee, but I can't eat it anymore." (FG2, P1)*
	**Challenges of health behaviours**	The difficulties in adhering to recommended health behaviours for T2DM treatment advice, including weight management, physical activity, and dietary compliance, and sheds light on the barriers that hinder the effective implementation of interventions	*"Losing weight is the big talk, but it's easier said than done, you know? …… The doctor told me to get moving, but honestly, I just can't be bothered." (FG2, P2)* *"The doctor advised me to increase my physical activity, but I struggle with that. I really should tell the doctor about my knee pain!" (FG1, P5)*
**Personal resources**	**Expenses**	The financial challenges of T2DM treatment, covering direct medical costs and indirect expenditures such as medications, monitoring, consultations or self-management supplies	*"Four injections per month cost me around 2000 yuan. On a salary of just 3,000 to 4,000 yuan, that's a huge financial burden." (FG3, P5)* *"Money is tight. It's not just the medicine, but also all the checks and tests I have to pay for." (FG1, P5)* *(Some other participants questioned why he still found the burden heavy despite having retirement medical insurance covering 85% of costs.) "Sure, hospital stays get covered, but the outpatient clinic? Not a dime. After retirement, my only income gone." (FG2, P1)* *"The testing strips are the worst. The ones I use cost 315 yuan for 50. I need to test three times a day, so they don't last long." (FG1, P3)* *"My glucose levels have been inconsistent, but skipping expenses for children's education wasn't an option. It's becoming a financial strain." (FG4, P2)*
	**Time**	The substantial time commitment necessary for treatment task, including daily medication or insulin administration, waiting for healthcare services, and routine monitoring	*"It's a cumbersome process. Going through the pharmacy in clinic takes a lot of time, it's exhausting." (FG1, P2)* *"I've been advised to stick to my medication and keep track of my health metrics in clinic. But honestly, every trip for a check-up is a hassle, what with the long waiting." (FG4, P2)* *"As for getting registered, there's a sea of people waiting. The lines are just too long, eats up a lot of time." (FG3, P5)*
	**Travel**	The logistical hurdles in accessing healthcare, such as the distance to medical facilities and the added complications brought about by external factors like the pandemic	*" Sometimes, I have to go quite a distance just to pick up my medicine." (FG4, P4)* *"My friend's medical insurance is tied to his hometown (in another province). He has to travel back just to get his medicine. How tricky it can be, to manage diabetes when you're away from home." (FG4, P4)* *"(During pandemic) If I want to take public transport, I need a RATs (Rapid Antigen Tests) result from the last 24 h." (FG1, P1)*
**Associated factors**	**Antecedents**	This category encompasses underlying determinants such as health literacy, health locus of control, comorbidities, and socioeconomic status, which influence the individual’s perception of T2DM treatment burden. It focuses on patient or disease characteristics that precede the experience	*"Since I was a nurse, I grasp these medical instructions with ease. However, I realise that for someone without medical knowledge, deciphering complex instructions and ensuring correct medication intake could be quite challenging." (health literacy, FG1, P4)* *(Patient’s insight of why feeling stressful of the administrative task) "I feel like managing my diabetes is entirely in my hands. If my blood sugar spikes, it might because I didn't follow the diet or exercise plan properly." (health locus of control, FG 4, P3)* *(Patient with a hearing impairment): "It’s tough getting what the doctors said. I've got to use my phone or hearing aids just to get by in a simple chat." (comorbidities, FG1, P1)* *"After my thyroid cancer surgery in '04, I've been down to one vocal cord. Makes it hard to talk, especially with healthcare folks. And the pills! Three for the thyroid and six for my diabetes, every single day. It's just overwhelming." (comorbidities, FG1, P2)*
	**Consequences**	This category outlines the downstream outcomes of T2DM treatment burden. It specifically addresses the resultant impacts on adherence to treatment, psychological well-being, social life, personal function and overall quality of life due to the treatment burden	*"I worry about the future. A single child may bear the treatment responsibilities for four or six elders. When illness hits most of us, who will shoulder mine (the treatment)?" (psychological distress, FG3, P2)* *"Whenever I'm out with colleagues, I have to sneak off to manage my diabetes, typically to administer my insulin. This behaviour can make me seem peculiar, and frankly, it's not something I feel like having to explain." (challenges in social and interpersonal, FG4, P1)* *"Because of my dietary needs, the whole family has shifted towards eating coarse grains. Although they haven't complained, I know it has affected our quality of life." (quality of life and challenges in social and interpersonal, FG3, P6)*

### Theme 1: Medical information

This theme underscores the challenges faced by individuals with T2DM in accessing, understanding, and trusting medical information. It reflects the cognitive burden stemming from complex medical terminologies and the emotional impact linked to difficult-to-comprehend information. Moreover, this theme delves into the credibility and potential source-specific bias of medical information. Within this theme, three critical sub-themes emerged. "Cumbersome Medical Information" refer to the complexity and user-unfriendliness of medical information presentation. "Lack of Information Sources" highlights the struggle to find consistent and personalised medical information. "Biased Information" highlights the impact of cultural and societal biases on the interpretation of medical information.

Our analysis revealed a substantial burden for individuals with T2DM in managing medical information. Participants frequently cited difficulties in comprehending complex medical details and choosing from various treatment options. This complexity exacerbates the challenge of understanding and managing T2DM, leading to an increased treatment burden. For example, one participant from a rural focus group (FG4, P1) expressed confusion over the implications of high blood sugar levels, despite undergoing multiple tests. Another participant (FG4, P2) mentioned being overwhelmed by the wide range of available therapies for the same condition. The scarcity of reliable information sources further aggravates these challenges, causing uncertainty and confusion. Furthermore, cultural and societal influences notably shape how medical information is interpreted, as evidenced by misconceptions about insulin-causing hypoglycaemia (FG1, P3) and certain dietary practises (FG3, P2).

### Theme 2: Healthcare system

This theme delves into the structural and functional challenges within the healthcare system that amplify the treatment workload for individuals. It covers systemic issues affecting healthcare delivery and efficiency. The theme is further broken down into four sub-themes. "Healthcare Fragmentation" highlights the difficulties individuals encounter in navigating a segmented healthcare system and coordinating treatment across multiple departments and providers. "Healthcare Provider" refer to the intricate challenges in patient-provider interactions, this sub-theme highlights the limitations in consultations and the impact of healthcare providers' attitudes. "Insurance or Resource Use" reflects the complexities and inconsistencies in health insurance coverage and public resource utilisation are discussed, emphasising the additional effort needed for tasks like reimbursement claims. "Difficulty with Healthcare Access” highlights the systemic barriers to treatment accessibility, including internal restrictions within medical and insurance systems, and external factors like the COVID-19 pandemic.

Participants identified healthcare fragmentation as a substantial challenge stemming from systemic inadequacies in the primary care system. This issue was particularly pronounced among participants from remote and rural areas (FG3, P2; FG4, P2), who struggled with inconsistent advice and navigating multiple departments. Challenges with healthcare providers, such as limited resources and variable attitudes, further complicated treatment experiences, impacting individuals' confidence and adding stress to healthcare visits (FG1, P4; FG3, P5). Complexities in insurance coverage, including inconsistent reimbursements and bureaucratic hurdles, were also notable concerns (FG4, P4). Moreover, the accessibility of healthcare services, exacerbated by external factors like pandemics, presented additional hurdles for patients (FG1, P2).

### Theme 3: Administration

This theme addresses the management of various medical and non-medical tasks that are integral to effective T2DM treatment. These tasks, while essential, can considerably increase the workload for individuals. The theme is broken down into several distinct sub-themes. "Periodic Examination/Monitoring" encompasses the challenges related to regular medical check-ups and the resource-intensive nature of ongoing monitoring, which can be particularly demanding in terms of time and physical effort. "Arranging Appointments" highlights the difficulties encountered in scheduling medical visits, a task that can be especially challenging for older individuals who may find it hard to navigate technological systems. "Documentation and Paperwork" focuses on the complexities involved in maintaining accurate health records and managing necessary documentation, which can be overwhelming and confusing.

Notably, "Glucose Meters" sub-theme represents a distinctive aspect of diabetic administration, focusing on the specific challenges associated with the use of glucose meters. This sub-theme is characterised by issues related to their frequent use, the accuracy of readings, and the physical discomfort they may cause. While there is a potential overlap with other sub-themes within "Administration", this area warrants particular attention due to its unique contribution to measuring and inquiring about the treatment burden in T2DM care [43]. A thorough analysis, further supported by discussions with experts and input from the PPIE panel, has underscored the necessity of retaining "Glucose Meters" as an independent sub-structure. This decision recognises the specific challenges these devices present in the management of diabetes. This approach is consistent with the decision-making process applied to the "Insulin- or Injection-Related Burden" sub-theme below.

### Theme 4: Medication

This theme explores the multifaceted challenges surrounding medication, addressing both practical and psychological aspects. It encompasses a broad spectrum of issues ranging from medication adherence and management to navigating side effects and complex treatment regimens. The theme is dissected into several sub-themes. "Management of Medications" captures the logistical challenges encountered, such as medication storage and scheduling. "Complexity of Medication Use" highlights the burden associated with managing multiple medications, including traditional medicines. "Ambivalence towards Medication" reflects the conflicting feelings towards medication use, where necessity is weighed against concerns over dependency.

The "Insulin- or Injection-Related Burden" is one of the emerging constructs in our analysis. After analysis and discussions with experts and the PPIE panel, this sub-theme has been retained as an independent sub-structure, as it reflects the unique challenges in T2DM therapies. Conversely, the "Side Effects and Hypoglycaemia" sub-theme refers to the discomfort and additional healthcare interactions caused by medication. The emerging construct of "Medication-related Hypoglycaemia" was merged with "Side Effects" into this single sub-theme. This decision was influenced by input from the PPIE panel, which underscored the common difficulty in distinguishing between side effects and hypoglycemics episodes within China’s primary care settings.

### Theme 5: Lifestyle

This construct highlights the impact of T2DM treatment on individuals' daily lives, particularly focusing on the disruption of lifestyle and daily routines as well as the behavioural challenges linked with treatment. The "Interruption of Lifestyle and Daily Routines" sub-theme highlights the lifestyle changes necessitated by T2DM treatment, such as dietary adjustments and changes in leisure activities, emphasising the conflict between personal lifestyle choices and the compromises required by the disease. "Challenges of Health Behaviours" refers to the difficulties in adhering to recommended health behaviours for T2DM treatment advice, including weight management, physical activity, and dietary compliance, and sheds light on the barriers that hinder the effective implementation of interventions.

### Theme 6: Personal resources

**Fig. 1:**
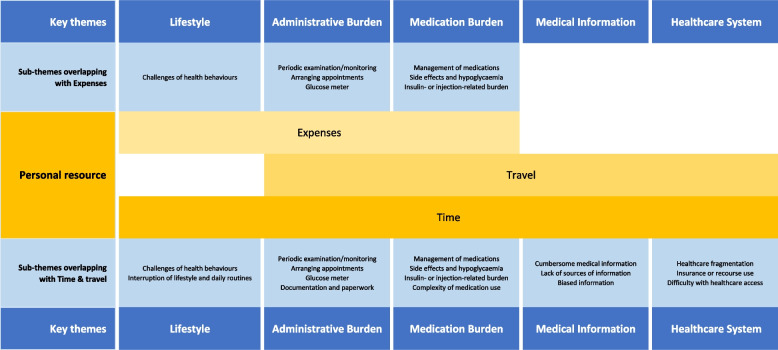
Illustrates the overlaps among sub-themes of key themes and sub-themes in personal resources, as identified and grounded on the qualitative data collected

In Theme 6, the focus group discussions brought to light how finance, time, and travel are frequently intertwined with other themes (Fig. 1). Discussions about the financial burden often co-occurred with sub-themes in administration, medication, and lifestyle burdens. Similarly, concerns about time and travel emerged alongside medical information, administration, and healthcare system issues. Drawing from the Normalisation Process Theory (NPT) [44], it is evident that treatments demand substantial personal investment in terms of energy, resources, and finances for specific behaviours and tasks. The constructs of financial, time, and travel burdens, key to this personal investment in treatment, are not seen by participants as isolated challenges. Instead, they are perceived as interconnected elements of their overall treatment experience. This observation aligns with a cognitive tendency known as contextual thinking, wherein individuals naturally integrate various aspects of their experiences [45]. This holistic perspective, recognising the mutual influence of different treatment burden factors, leads to the conceptualisation of "Personal Resources". This theme, which encapsulates finance, time, and travel, underscores the overlapping nature of these burdens and views them as interrelated rather than independent entities.

The "Expenses" sub-theme informs the financial challenges, underscoring the impact of both direct medical costs and indirect expenses associated with self-management and health behaviours. "Time" informs the substantial time commitment necessary for treatment tasks, including daily medication or insulin administration, waiting for healthcare services, and routine monitoring. "Travel" informs the logistical hurdles in accessing healthcare, such as the distance to medical facilities and the added complications brought about by external factors like the pandemic.

### Development of sub-themes

In the process of thematic analysis, researchers meticulously evaluated terminology that was initially derived directly from existing literature. Emerging evidence from the focus groups necessitated the development of several sub-themes, both in terms of terminology and descriptions, to more accurately reflect the content of the constructs identified. Specific revisions were made as follows: "drug dependence" was redefined as "ambivalence towards medication"; similarly, "challenges of medical regimen" was redefined as "periodic examination/monitoring"; "difficulty navigating the health system" was redefined as "difficulty with healthcare access"; “change of nature behaviour” was redefined as “interruption of lifestyle and daily routines”. The revisions undertaken are aimed at more accurately representing the specific subconstructs within the theme and distinguishing them from other sub-themes. Detailed definitions and illustrative quotes for these revised sub-themes are presented in Table 4.

### Associated factors

In exploring the participants experience on T2DM treatment burden, this study has identified several associated factors that extend beyond the key construct themes. These factors, which include antecedents and consequences, frequently surfaced in participant discussions and are integral to understanding the holistic treatment burden. However, it is crucial to note that these factors do not directly represent the perceived “workload” of treatment or the individual’s “capacity” to manage this workload. Instead, they offer supplementary insight into the broader context of how individuals perceive and experience T2DM treatment burden.

The "Antecedents" theme encompasses underlying factors such as health literacy, health locus of control, comorbidities, and socioeconomic status, which shape an individual’s perception of T2DM treatment burden. While these antecedents do not directly form the core construct of T2DM treatment burden, they are pivotal in understanding the preceding patient or disease characteristics that influence the treatment experience. Similarly, the theme of "Consequences" outlines the downstream outcomes of T2DM treatment burden, including adherence to treatment protocols, psychological distress, quality of life, and challenges in social and interpersonal relationships. The consequences factors illuminate the wide-ranging repercussions of the treatment burden on individuals' lives.

## Discussion

 This study offers a qualitative insight into the treatment burden of T2DM within primary care settings in China. The CuCoM elucidates the interaction pattern between an individual’s treatment workload and their capacity in terms of physical, emotional, and social resources [10]. According to this pattern, our analysis identifies five key constructs of T2DM treatment burden mentioned by patients. The treatment of T2DM places a substantial workload on individuals, encompassing various aspects such as managing medical information, navigating the healthcare system, medication adherence, administrative tasks, and lifestyle adjustments. Previous studies have incorporated the burdens of administration, medication, and lifestyle within multiple theoretical frameworks and measurement instruments [7–9]. In our study, the focus group data serves to refine these themes, enhancing their application in interrelating the treatment burden of T2DM.

Additionally, the focus group's emphasis on medical information highlights a gap in current research and treatment burden measurement, particularly the under-representation of cognitive and emotional aspects in managing T2DM-related medical information [46]. In previous generic measurement for treatment burden, only a few have mentioned this dimension; moreover, due to a lack of related research, it is challenging to refine an effective measuring approach [31, 47]. The final framework provides an interpretive approach for understanding these experiences, aiding health professionals in effectively identifying specific T2DM treatment burdens in various primary care settings [23].

On the other hand, previous studies have proposed redefining "treatment burden" over "workload", describing it as encompassing both direct treatment workload and its impact on daily life, including work, social, and caring responsibilities [48]. This perspective illuminates how factors of burden and the interaction of resources with healthcare utilisation influence an individual’s engagement with treatment and their experience of burden [11]. Building on this definition, May et al. introduced the NPT as an appropriate framework for analysing treatment burden [49]. The integration of NPT with the CuCoM establishes a robust theoretical foundation, facilitating a deeper exploration of the multifaceted complexity of the burden and resources allocation and utilisation in T2DM treatment [11, 48].

This analysis led to the introduction of the "personal resources" concept, which presents overlapping nature with other constructs in the conceptual framework. Previous studies and measurements often considered the financial aspect as a distinct dimension of treatment burden [12, 50]. However, our focus group findings reveal that participants perceive financial, time, and travel burdens not as isolated challenges, but as interconnected components of their experience with other aspects of treatment burden. The findings demonstrate the contextual thinking in participants, where individuals naturally combine various facets of their treatment burden with the resource constraints they faced [45]. This tendency is particularly apparent in primary care context. Recognising these interconnections makes the framework more relevant to real-world T2DM care in these settings. It highlights the need for a holistic approach to personal resource allocation and utilisation, embracing both tangible and intangible aspects, to thoroughly understand and effectively tackle the complexities of treatment burden.

In this study, while identifying themes and key constructs relevant to the T2DM treatment burden, we also place emphasis on associated factors, namely antecedents and consequences. These factors, though not directly quantifying the perceived "workload" of treatment or the individual's "capacity" to handle this workload [10], contribute significantly to a more comprehensive understanding of T2DM treatment burden as observed in the focus group discussions. Antecedents shed light on individual vulnerability, influencing how patients perceive and manage their treatment burden, while consequences offers insight into the long-term management of treatment burden [31, 51]. This approach aligns with research by Sav et al., which articulates the interplay of these factors in treatment burden and suggests a cyclical relationship between antecedents and consequences [12]. From a measurement perspective, patient and treatment experiences could be evaluated using specific patient-reported outcome measurements [52]. Recognising the potential for patient outcome measurements and interventions targeting these aspects offers potential to positively influence treatment burden. This finding underscores the necessity of simultaneously measuring and addressing these associated factors in treatment burden research.

### Limitations

The qualitative methodology and thematic analysis provided in-depth insights into the treatment burden experienced by people with T2DM in China’s primary care settings. The development of themes corresponds with our earlier findings from a retrospective analysis of qualitative data obtained from clinical consultations. Despite structural adjustments, the stability and observable nature of these key constructs have been affirmed [47].

In alignment with the primary aim, a priori thematic saturation was prioritised to ensure the broader applicability of the findings [53]. An initial narrative review established a coding structure based on existing evidence (Table 1), which facilitated an in-depth exploration during the focus group discussions and data analysis [22]. Participants for the focus group were carefully chosen to provide a diverse representation of healthcare contexts while maintaining demographic homogeneity, consistent with the narrowly defined objective of this study. This approach led to the achievement of a priori thematic saturation [41]. In addition, the range of sample sizes for focus group typically spans from 4 to 8, which is generally consistent and sufficient to approach data saturation [41]. However, it is important to acknowledge that conducting a single focus group in each region does pose certain limitations. Specifically, this approach may not allow for achieving data saturation in each region to the extent required for a detailed comparative analysis [33]. Further research could build on our findings by conducting more extensive investigations in specific regions. This would enable a deeper and more nuanced comparative analysis of the themes emerging from different settings, thereby enriching our understanding of the subject matter.

An additional concern is that general practitioners in the clinics assisted in the process of distributing flyers, which might have influenced participants' contributions to the discussions [20]. This is particularly relevant for sensitive topics that are closely related to the participants' own context, such as the healthcare system. This concern was noted in the reflective materials of one researcher, who observed that "one participant tended to excessively praise his general practitioner".

## Conclusions

The findings of this study represent a valuable contribution to the understanding of the T2DM treatment burden. We have developed a conceptual framework from patient perspectives, offering an in-depth overview of this burden. This framework, including key constructs that highlight the multifaceted nature and impact of treatment burden on individuals with T2DM. It also emphasises the importance of tailoring the treatment workload to the individual’s capacity, considering their personal resource allocation and treatment utilisation.

## Data Availability

The authors confirm that the data supporting the findings of this study are available within the article. The datasets analysed are available from the corresponding author, upon reasonable request.

## Abbreviations

CuCoM: Cumulative Complexity Model
FG: Focus Group
NPT: Normalisation Process Theory
PPIE: Patient and Public Involvement and Engagement
T2DM: Type 2 Diabetes Mellitus
TBQ: Treatment Burden Questionnaire
